# Regional Dichotomy in Enteric Mucosal Immune Responses to a Persistent *Mycobacterium avium* ssp. *paratuberculosis* Infection

**DOI:** 10.3389/fimmu.2020.01020

**Published:** 2020-05-29

**Authors:** Antonio Facciuolo, Amy H. Lee, Patricia Gonzalez Cano, Hugh G. G. Townsend, Reza Falsafi, Volker Gerdts, Andrew Potter, Scott Napper, R. E. W. Hancock, Lucy M. Mutharia, Philip J. Griebel

**Affiliations:** ^1^Vaccine & Infectious Disease Organization—International Vaccine Centre, University of Saskatchewan, Saskatoon, SK, Canada; ^2^Department of Microbiology and Immunology, Centre for Microbial Diseases and Immunity Research, University of British Columbia, Vancouver, BC, Canada; ^3^Department of Pharmacobiology, Universidad de la Cañada, Oaxaca, Mexico; ^4^Department of Biochemistry, Microbiology and Immunology, University of Saskatchewan, Saskatoon, SK, Canada; ^5^Department of Molecular & Cellular Biology, University of Guelph, Guelph, ON, Canada; ^6^School of Public Health, University of Saskatchewan, Saskatoon, SK, Canada

**Keywords:** bovine, small intestine, Mycobacterium, paratuberculosis, Peyer's patches, mucosal, IL22, IL27

## Abstract

Chronic enteric *Mycobacterium avium* ssp. *paratuberculosis* (MAP) infections are endemic in ruminants globally resulting in significant production losses. The mucosal immune responses occurring at the site of infection, specifically in Peyer's patches (PP), are not well-understood. The ruminant small intestine possesses two functionally distinct PPs. Discrete PPs function as mucosal immune induction sites and a single continuous PP, in the terminal small intestine, functions as a primary lymphoid tissue for B cell repertoire diversification. We investigated whether MAP infection of discrete vs. continuous PPs resulted in the induction of significantly different pathogen-specific immune responses and persistence of MAP infection. Surgically isolated intestinal segments in neonatal calves were used to target MAP infection to individual PPs. At 12 months post-infection, MAP persisted in continuous PP (*n* = 4), but was significantly reduced (*p* = 0.046) in discrete PP (*n* = 5). RNA-seq analysis revealed control of MAP infection in discrete PP was associated with extensive transcriptomic changes (1,707 differentially expressed genes) but MAP persistent in continuous PP elicited few host responses (4 differentially expressed genes). Cytokine gene expression in tissue and MAP-specific recall responses by mucosal immune cells isolated from PP, lamina propria and mesenteric lymph node revealed interleukin (*IL)22* and *IL27* as unique correlates of protection associated with decreased MAP infection in discrete PP. This study provides the first description of mucosal immune responses occurring in bovine discrete jejunal PPs and reveals that a significant reduction in MAP infection is associated with specific cytokine responses. Conversely, MAP infection persists in the continuous ileal PP with minimal perturbation of host immune responses. These data reveal a marked dichotomy in host-MAP interactions within the two functionally distinct PPs of the small intestine and identifies mucosal immune responses associated with the control of a mycobacterial infection in the natural host.

## Introduction

Johne's disease is a chronic, enteric infection of ruminants caused by *Mycobacterium avium* ssp. *paratuberculosis* (MAP). MAP is endemic worldwide (1) with high herd prevalence among Canadian dairy cattle (2), sheep and goats (3). The majority of MAP-infected cattle are asymptomatic (4) but infection results in significant economic losses (5) due to decreased milk production (6–8) and decreased slaughter value (9, 10). During the prolonged asymptomatic stage of infection animals intermittently shed MAP in feces (11) facilitating horizontal transmission from cow to calf (12, 13) and among calves (14, 15). MAP shedding in colostrum and milk (16) permits vertical transmission (17). Detection of MAP in the environment (18), drinking water (19), and retail milk (20) has led to concerns regarding food safety and the potential for MAP to further exacerbate human Crohn's and other autoimmune diseases (21–23).

The prolonged asymptomatic nature of MAP infection has hampered studies of naturally-infected cattle in early stages of infection (<1–2 y post-infection) since diagnostic tests are unreliable at this stage of infection (24). Diagnostic methods are more sensitive in identifying subclinical and clinical stage animals (2–5 y post-infection) (25) confining most studies of naturally infected cattle to this latter cohort. The absence of biomarkers that identify recently infected cattle has led to the development and use of animal models in which infectious dose and time post-infection can be clearly defined, facilitating analyses of initial host-pathogen interactions and early host immune responses. The use of temporary ligation of intestinal segments in calves (26) and goats (27) has contributed to an initial understanding that MAP invades the intestinal epithelial barrier via M-cells overlying Peyer's patches (PP), leading to the immediate uptake and persistence of MAP in subepithelial macrophages. Oral inoculation challenge models (28, 29), tonsillar crypt instillation (30) and ileal cannulation models (31) revealed that systemic host immune responses can occur as early as 3 months post-infection and continue through to 9 months post-infection. Few studies have analyzed the mucosal immune responses occurring at the site of infection in cattle. Global transcriptional changes in ileal tissue were identified at 12 h post-infection (32). Intraepithelial lymphocyte activation and differential cytokine gene expression were identified in ileal tissue at 6–9 months post-infection (31) and draining mesenteric lymph node (MLN) cells at 7 and 15 months post-infection (33).

We developed surgically isolated intestinal segments in neonatal calves as a model to investigate early (1–2 months post-infection) (34) and persistent (9–11 months post-infection) (35) MAP infections. Similar to previous models, we utilized the natural host and targeted delivery of a defined dose of MAP to specific sites in the small intestine. Advantages of the surgical isolation model include delivery of a defined dose of MAP to a localized site of infection and prevention of the spread of infection to adjacent intestinal tissues. MAP shedding in feces can occur days, weeks, and months following oral MAP challenge (36, 37), possibly re-infecting adjacent intestinal tissues. Recurrent enteric infections could potentially induce additional mucosal immune responses and increase trafficking of effector lymphocytes to the initial site of infection. Our model permits a more consistent challenge among individuals in an out-bred species. Using surgically isolated intestinal segments, we first reported a significant dichotomy in mucosal antibody responses when comparing MAP infection of bovine discrete PP (DPP; distributed throughout the jejunum) vs. continuous PP (CPP; restricted to the terminal jejunum and ileum) (34). This divergence in host mucosal immune responses has not been investigated in naturally infected nor experimentally infected cattle but has been studied in goats and sheep (38–40).

In ruminants, 25–40 DPPs, also referred to as jejunal PPs, are dispersed throughout the jejunum and function as mucosal immune induction sites (41). The DPPs sample luminal antigens and generate IgA B cells in an antigen-dependent manner via cognate interactions with CD4^+^ T cells present within the submucosal lymphoid follicles (34, 42, 43). The CPP, also referred to as the ileal PP, occupies the terminal 1–3 m of the small intestine of young calves and terminates at the ileocecal junction. The CPP functions as an antigen-independent primary lymphoid organ for diversification of the immunoglobulin repertoire of naïve B cells. This primary lymphoid tissue is characterized by expression of activation-induced cytidine deaminase during fetal development (44), a paucity of CD4^+^ T cells in lymphoid follicles (41, 42, 45) and the emigration of sIgM^+^ B cells to all secondary lymphoid tissues (46). We previously demonstrated that MAP could infect both types of PPs in young calves and that infection of DPP, but not CPP, induced MAP-specific IgA B cell responses at 2 months post-infection (34). The absence of MAP-specific antibody responses in CPP is consistent with antigen-independent B cell development in this tissue.

Mucosal immune responses to MAP infection in cattle have not been thoroughly investigated, with most studies having focused on CPP. Our previous report (34) was the first to analyze mucosal immune responses in bovine DPP. Detection of MAP in DPP has been reported in both naturally infected (47, 48) and experimentally infected cattle (30, 31, 36, 49–51). However, the significance of infection in DPP vs. CPP and the contribution of DPP to Johne's disease in cattle has been largely ignored. Understanding host-pathogen interactions in DPP is critical for a complete understanding of MAP pathogenesis and the role of mucosal immune responses throughout the small intestine. If MAP infection of DPPs, a major mucosal immune induction site, results in control of bacterial burden then analyzing these local immune responses may provide insight into the immune responses required to control MAP infection. Furthermore, differential responses in DPPs vs. the CPP may provide information regarding the mechanisms contributing to asymptomatic infections and/or immune reactivity and pathology.

Considering the prolonged host-pathogen interaction that occurs between pathogenic *Mycobacterium* species and their hosts, we re-examined the outcome of MAP infection in DPP when compared to CPP to investigate whether the induction of an adaptive immune response in DPP could progress to a protective response, unlike that previously reported for the CPP (35). To address this, a defined dose of MAP was targeted to surgically isolated intestinal segments containing either a DPP or CPP in neonatal calves (10–14 days). At 12 months post-infection, intestinal segments were collected and a systems biology approach was used to analyze global transcriptional changes (RNA-seq) in infected vs. uninfected DPP and CPP. Mucosal immune responses were further investigated by analyzing cytokine gene expression in intestinal tissue and in isolated mucosal immune cells re-stimulated with MAP antigen. RNA-seq analyses revealed marked differences between DPP and the CPP with an inverse relationship between MAP persistence and dysregulation of the host transcriptome. Subsequent qRT-PCR analysis identified differentially expressed cytokine genes in both DPP and CPP tissues. Re-stimulation of immune cells isolated from the intestinal lamina propria, submucosa and draining MLNs with MAP antigen revealed that differential (*p* < 0.05) expression of *IL22* and *IL27* was associated with the control of MAP infection. This comparative analysis of host responses in DPP and CPP provides novel insight into immune responses associated with the control of MAP and identifies possible correlates of immune protection.

## Materials and Methods

### Ethics Approval Statement

All experiments were completed at the University of Saskatchewan following regulations established by the Canadian Council on Animal Care and approved by the University of Saskatchewan Animal Care Committee (Protocol #20160076).

### Animals, Surgery, and MAP Infection

Ten- to fourteen-day old healthy, male Holstein calves were purchased from a local supplier. Housing, feeding, anesthesia, surgery, post-surgical care, and MAP infection have been previously described in detail (34, 52). Calves were individually housed until 6 weeks of age and then group housed until the end of the trial. No other cattle were present in the facility throughout the study and localization of MAP infection to challenged intestinal segments precluded fecal shedding of MAP and environmental contamination (34, 35). Therefore, this model does not include a component of re-infection beyond the persistence of MAP in the contents of intestinal segments for at least 2 months post-challenge. As with all past and present surgeries, special care was taken to preserve vasculature and lymphatic connections of each intestinal segment through the mesentery attachment and continuity of the intestinal tract was re-established by an end-to-end anastomosis of the intestine proximal and distal to the intestinal segment. Two preliminary studies were completed to confirm the consistency and reproducibility of our bovine enteric infection model using the MAP strain gc86. This strain is a low-passage Type II field isolate of MAP (53) and has previously been used for calf infection studies (34, 54). The first study was to validate that our infection model results in equal uptake and persistence of MAP strain gc86 in DPP and CPP early in infection. Two intestinal segments, one in the mid-jejunum containing a DPP and the other proximal to the ileocecal fold (i.e., terminal jejunum) containing a CPP were surgically isolated in 10–14 day old calves (*n* = 5) and 1 × 10^9^ MAP colony-forming units (CFU) of strain gc86 injected into the lumen. At 28 days post-infection PP tissue was harvested, homogenized and serial dilutions plated (see *MAP Detection*) to enumerate MAP CFU (Supplementary Figure 1A). The second study was to establish that our model results in consistent and reproducible infection in all intestinal segments. In three independent trials (Trial 1: *n* = 11; Trial 2: *n* = 17, Trial 3: *n* = 18) intestinal segments containing a DPP were surgically isolated in 10–14 day old calves and 1 × 10^9^ CFU MAP strain gc86 injected into the lumen. At 28 days post-infection PP tissue was harvested, homogenized, and serial dilutions plated to enumerate MAP CFU (Supplementary Figure 1B).

To address the central focus of this study, 15 calves were randomly assigned to one of three groups receiving the following treatment: (1) Surgical isolation of a single 15 cm intestinal segment in the mid-jejunum containing a DPP; (2) Surgical isolation of a single 15 cm intestinal segment proximal to the ileocecal fold (i.e., terminal jejunum) containing CPP; and (3) Surgical isolation of two 15 cm intestinal segments, one containing a DPP and the other a CPP. Immediately following surgical isolation, 1 × 10^9^ CFU MAP strain gc86 suspended in 5 mL calcium- and magnesium-free phosphate-buffered saline [PBS] was injected into the lumen of intestinal segments of Group 1 and 2 calves. Five mL of PBS was injected into the lumen of intestinal segments in Group 3 calves.

### Gross Examination and Histology

At 12 months post-surgical isolation and infection of intestinal segments, calves were euthanized by intravenous injection with Euthanyl (20 mL/45 kg body weight; Bimeda-MTC, Canada) and tissue collected within 10–15 min. Intestinal segments, adjoining intact intestine and MLN were collected and photographed to record gross appearance. Intestinal segments were opened along the mesenteric attachment, luminal contents collected, and the appearance of the mucosal surface examined, photographed, and recorded. Tissues were immediately immersed in 10% neutral-buffered formalin for histology. Tissue embedding, sectioning, hematoxylin and eosin (H&E) staining, and immunohistochemical staining (Ki-67 and MAP antigen) were completed by Prairie Diagnostic Services (Saskatoon, SK, Canada). Histological images were acquired using an Olympus Virtual Slide Scanning Microscope (Olympus-Life Science, Japan) and completed by the WCVM Imaging Center (University of Saskatchewan, Saskatoon, SK, Canada).

The length of individual lymphoid follicles within the CPP was measured to determine if isolating the PP within intestinal segments had a significant effect on follicular development when compared to CPP in the adjacent, intact intestine. Five lymphoid follicles were measured within each H&E stained tissue section of a CPP collected from the intestinal segment and the adjacent small intestine of four animals. Only follicles with a visible dome region were selected to ensure each measurement was made near a mid-sagittal section of the lymphoid follicle and the length of each submucosal follicle was measured from the follicle base to its junction with the muscularis mucosa. An ocular micrometer was used to measure follicle length and the ocular micrometer was calibrated using a 2 mm graticule with 0.01 mm intervals (Ernst Leitz GMBH, Wetzlar, Germany).

### MAP Detection (*hspX* and *f57* qPCR, and CFU Enumeration)

Detection of MAP in paraffin-embedded tissues sections was performed by Prairie Diagnostic Services. Briefly, DNA was extracted using the DNeasy Blood and Tissue Kit (Qiagen, Inc.) and the *hspX* gene amplified using the VetAlert™ Johne's Real-Time PCR kit (Tetracore Inc., USA).

For quantification of the single copy *f57* DNA element, PP tissue (minimum 50 mg) was collected, weighed and washed with three exchanges of PBS vortexing tissue samples each time for 20 s. The tissue was then homogenized in ATL buffer (Qiagen, Inc.) with 2.0 mm Zirconia beads in a Mini-Beadbeater-16 (Bio Spec Products Inc.). DNA was isolated from the clarified tissue supernatant using the DNeasy Blood and Tissue kit (Qiagen, Inc.) following the manufacturer's instructions. Genomic DNA was similarly isolated from MAP strain gc86 cells grown in broth culture (see *Preparation of MAP whole cell lysate*). DNA quality and quantity was determined using a NanoDrop™ Spectrophotometer (Thermo Fisher Scientific, Inc.) and gel electrophoresis. Real-time qPCR reactions, performed in duplicate, contained PerfeCTa SYBR Green SuperMix (Quanta Biosciences, Inc.), 300 nM of *f57* primers (55), and 50 ng of extracted DNA. Cycling conditions were initial denaturation for 3 min at 95°C followed by 40 cycles of 95°C for 15 s, 60°C for 30 s, and 72°C for 30 s using a Bio-Rad CFX Connect Real-Time PCR Detection System (Bio-Rad Laboratories, Inc.). Quantitative threshold cycle (Cq) for each reaction was determined by CFX Manager™ Software (Bio-Rad Laboratories, Inc.), and average Cq calculated using arithmetic average of duplicate reactions. Concurrently with all samples, a six 10-fold dilution series using purified MAP genomic DNA representing 1 × 10^6^ to 1 × 10^0^ genomic copies (6 ng to 6 fg, respectively, based on a genome size of 4.83 Mb) was amplified using the same cycling program to generate a standard curve to extrapolate gene copies in each sample. Data are expressed as *f57* gene copies per g of PP tissue. A preliminary study was completed to determine the correlation between *f57* gene copies and MAP CFU using *in vivo* infected tissue samples (Supplementary Figure 2). Intestinal segments containing a DPP were surgically isolated in 10–14 day old calves (*n* = 17) and 1 × 10^9^ CFU MAP strain gc86 injected into the lumen. At 28 days post-infection, DPP tissue was harvested, homogenized, and serial dilutions plated to enumerate MAP CFU. Homogenized samples were further subject to a low speed spin (10 min at 500 × g) to pellet cellular debris followed by a high-speed spin (5 min at 8,000 × g) to pellet MAP bacteria. DNA was isolated from this pellet following the extraction protocol for tissue samples described above.

For detection of viable MAP, PP tissue (minimum 100 mg) was collected, weighed and washed with three exchanges of PBS vortexing tissue samples each time for 20 s. Tissue was then homogenized in 5 mL PBS using a PRO200 homogenizer (PRO Scientific Inc., USA). Serial dilutions were plated on Difco™ Mycobacteria 7H11 Agar (BD and Company, USA) supplemented with 10% BBL™ Middlebrook OADC Enrichment (BD and Company, USA), 2 mg/L ferric mycobactin J (Allied Monitor Inc., USA), 50 μg/mL carbenicillin (MilliporeSigma Canada Co.) and 10 μg/mL amphotericin B (MilliporeSigma Canada Co.), incubated at 37°C and monitored weekly (up to 8 weeks) for colony formation.

### RNA-seq

Total RNA was extracted using the RNeasy Plus Mini kit (Qiagen, Inc.) following the manufacturer's protocol from tissue samples (PP and MLN) collected immediately following euthanization and preserved in RNA*later* (Life Technologies Corp.). Quantification and quality assessment of total RNA was performed using an Agilent 2100 Bioanalyzer (Agilent Technologies, Inc.) and only samples with RNA integrity number (RIN) >8 were used for subsequent steps of mRNA enrichment using the NEBNext Poly(A) mRNA Magnetic Isolation Module (New England Biolabs, Inc.). Strand-specific cDNA libraries were generated from poly-adenylated mRNA using the KAPA Stranded RNA-Seq Library Preparation Kit (Roche Sequencing and Life Science). Adapters (Bio Scientific, USA) for multiplexing were ligated, followed by amplification and then purification using Agencourt Ampure XP beads (Beckman Coulter Life Sciences). The quality of the library was checked using a high-sensitivity DNA chip (Agilent Technologies, Inc.) on an Agilent 2100 Bioanalyzer. All cDNA libraries were prepared at the same time and all sequenced on the NextSeq (Illumina, Inc.) on two runs, a single-end run of 100 bp-long and a paired-end run of 2 x 75 bp-long sequence reads (+ adapter/index sequences).

After demultiplexing, FASTQ sequence quality was assessed using FastQC v0.11.6 and MultiQC v1.6. The FASTQ sequence reads were aligned to the *Bos taurus* genome ARS-UCD1.2 (Ensembl release 95.12) using STAR v2.6.1d and mapped to Ensembl release 95.12 transcripts. Read-counts were generated using htseq-count (HTSeq 0.11.2). Samples were sequenced to a medium sequencing depth of 12 million uniquely mapped reads (range of 2.3–56.7 million reads). All data processing and subsequent differential gene expression analyses were performed using R version 3.6.0 and DESeq2 version 1.24.0. Genes with very low counts (with <10 counts in eight or more samples, or the smallest number of biological replicates within each treatment group) were pre-filtered and removed *in silico*. Differentially expressed genes were identified with the Wald statistics test and filtering for any genes that showed 1.5-fold change and adjusted *p* ≤ 0.05 (cut-off at 5% FDR) as the threshold. Functional discovery of pathway enrichment and network analyses was performed using InnateDB (56) and NetworkAnalyst (57), respectively.

### RNA Extraction From Tissue and Cells

RNA was isolated from tissue and cells using the RNeasy Mini Kit (Qiagen, Inc.) with slight modifications. For each intestinal segment, PP tissue was excised immediately after euthanization, immersed in RNA*later* (Life Technologies Corp.) overnight at 4°C, and subsequently stored at −80°C. Samples were thawed and homogenized in RLT buffer (Qiagen, Inc.) containing 1% beta-mercaptoethanol for 2 × 20 s using 2.0 mm Zirconia beads in a Mini-Beadbeater-16 (Bio Spec Products, Inc.). RNA was isolated from the clarified supernatant as per the manufacturer's instructions. Control and MAP-infected DPP and CPP tissue archived from a previous study (34) were also processed in the current study. These samples were collected from intestinal segments prepared in 10–14 day old calves in which 1 x 10^9^ CFU MAP strain gc86 was injected into the lumen and PP tissue harvested at 2 months post-infection. PP tissue was preserved in RNA*later* at −80°C and RNA extracted as described above.

Cells lysed and preserved in TRIzol Reagent (Life Technologies Corp.) were extracted once with chloroform (0.2 mL/mL TRIzol Reagent) and RNA isolated from the aqueous phase as per the kit's instructions. Samples were stored at −80°C. RNA integrity, quality and quantity were assessed using an Agilent 2100 Bioanalyzer (Agilent Technologies, Inc.) and Nanodrop™ Spectrophotometer (Thermo Fisher Scientific, Inc.).

### Reverse Transcription and Real-Time qRT-PCR

One μg of RNA was pre-treated to remove contaminating genomic DNA and reverse transcribed using the QuantiTect Reverse Transcription Kit (Qiagen, Inc.) as per the manufacturer's instructions. After cDNA synthesis samples were diluted with RNase-, DNase-free water to a concentration of 10 ng/μL and stored at −20°C. Real-time qPCR reactions were set up in duplicate with each reaction consisting of PerfeCTa SYBR Green SuperMix (Quanta Biosciences, Inc.), 300 nM of gene-specific primers (Supplementary Table 1) and 50 ng of cDNA in a final volume of 15 μL. Thermal cycling program was 2 min at 95°C for initial denaturation followed by 36 cycles of 95°C for 15 s, 60°C for 30 s and 72°C for 30 s using a Bio-Rad CFX Connect Real-Time PCR Detection System (Bio-Rad Laboratories, Inc.). Quantitative threshold cycle (Cq) for each reaction was determined by CFX Manager™ Software (Bio-Rad Laboratories, Inc.), and average Cq calculated using arithmetic average of duplicate reactions. Five constitutively expressed genes [*ACTB, GAPDH, H3F3A, PPIA*, and *YWHAZ*; (58)] were assayed in all cDNA samples and analyzed using CFX Manager™ Software revealing *YWHAZ* and *PPIA* as most stably expressed based on coefficient of variance (0.182 and 0.114, respectively) and M values (0.45 and 0.39, respectively). For all tissue samples, the average Cq of *YWHAZ* and *PPIA* was applied as the reference standard to normalize Cq values. For *in vitro* re-stimulation assays with isolated mucosal immune cells, *YWHAZ* was used to normalize Cq values obtained from resting (i.e., medium alone) and stimulated cells, and relative expression calculated using 2^−ΔΔCq^ as previously described (59). For tissue samples, the mean ΔCq of the PBS control samples—for each gene of interest—was used as a baseline to subtract each individual PBS control and MAP-infected ΔCq value, and fold-change relative to the mean of PBS control samples calculated using the equation 2^−[baselineΔCq−sample(x)ΔCq]^.

### Primer Selection and Design

Gene-specific primers used in this study are listed in Supplementary Table 1. Specificity of published primer sets were validated by melt curve analysis and gel electrophoresis using cDNA derived from PP tissue and MLN. Primers developed in this study were designed using Primer3 [v.0.4.0; (60)] based on *Bos taurus* sequences obtained from NCBI. Specificity of PCR amplicons amplified from PP and MLN cDNA was validated by gel electrophoresis and melt curve analysis. PCR amplicons were cloned into pCR™2.1 vectors using the TA Cloning™ Kit (Life Technologies Corp.) and identity validated by sequencing. Five points of a 10-fold dilution series using linearized vectors containing the amplicon of interest were used to assess amplification efficiency of each primer set (E=10^(−1/slope)^). Amplification efficiency of all primer sets used was between 1.90 and 2.00.

### Isolation of Mucosal Immune Cells

A single lymph node (MLN) was located in the adjacent mesentery of each intestinal segment, collected in ice-cold HyClone™ Dulbecco's Low Glucose Modified Eagles medium (DMEM; GE Healthcare Life Science) immediately after euthanization, and cells isolated using the protocol previously described (34). MLN cells were suspended at 1 × 10^7^/mL in DMEM supplemented with 10% FBS plus antibiotics, antimycotic (penicillin, streptomycin, amphotericin B; MilliporeSigma Canada Co.) and 10 μg/mL gentamicin (“complete medium”). Similarly, following euthanization, each intestinal segment was dissected along the mesenteric border exposing the mucosal surface and rinsed with water. In intestinal segments containing a DPP, the PP tissue was excised along the visible margins and placed in ice-cold DMEM; the remaining non-PP mucosal tissue was collected in ice-cold Hanks-buffered saline solution (HBSS). For intestinal segments containing a CPP, a portion of PP tissue was collected in ice-cold DMEM and another in ice-cold HBSS. Tissue in DMEM was processed to collect cells in the submucosa, which include those in the lymphoid follicles and within the interfollicular regions, herein referred to as “PP cells” following a previously established protocol (34). Tissue in HBSS was processed to isolate lamina propria leukocytes (“LP cells”) as previously described (61). The resulting cell suspensions were adjusted to 1 × 10^7^/mL in complete medium. Viability of MLN, LP and PP cells was consistently > 98% as determined by trypan blue exclusion.

### *In-vitro* Re-stimulation Assay

LP, MLN, and PP cells (2 × 10^6^) were seeded in 12-well tissue culture plates in a final volume of 1 mL in complete medium. Cultures were stimulated with medium alone or one μg/mL MAP whole cell lysate (WCL) prepared in complete medium, cells were cultured at 37°C under 5% CO_2_ in a humidified chamber. At 24 h post-stimulation, cells in suspension were collected and centrifuged for 7 min at 300 × g, and 1 mL of TRIzol Reagent (Life Technologies Corp.) applied to each well to detach and lyse adherent cells. After centrifugation the supernatant was discarded and TRIzol Reagent from the corresponding well used to lyse the cell pellet. Samples were subsequently incubated at room temperature for 10–15 min and stored at −80°C.

### Preparation of MAP Whole Cell Lysate

A log-phase culture of MAP strain gc86 cultured in Difco Middlebrook 7H9 Broth supplemented with 10% Middlebrook OADC enrichment (BD and Company, USA) and 2 mg/L ferric mycobactin J (Allied Monitor Inc., USA) was used to prepare WCL. MAP cells were pelleted by centrifugation for 5 min at 12,000 × g, suspended in ice-cold lysis buffer [PBS, 5% glycerol, 5 mM EDTA, 1mM PMSF] and homogenized 5 × 25 s with 0.1 mm Zirconia/Silica beads using a Mini-Beadbeater-16 (Bio Spec Products Inc.). Protein concentration of the clarified supernatant was quantified using a Pierce BCA Protein Assay Kit (Thermo Fisher Scientific, Inc.) and aliquots stored at −20°C.

### Data and Statistical Analysis

Gene expression stability for reference genes in real-time qRT-PCR was evaluated using Bio-Rad CFX Manager™ Software. RNA-seq data analysis is described within its section. GraphPad Prism 8 (GraphPad Software, Inc., USA) was used for all other data visualization and statistical analyses. Assumptions of normal data distribution were tested using the Shapiro-Wilk normality test. Differences in cytokine transcript abundance in PP tissue between MAP-infected (DPP, *n* = 5; CPP, *n* = 4) and PBS control (DPP, *n* = 4; CPP, *n* = 4) intestinal segments was determined using an unpaired, two-tailed Student's *t* test and presented as fold change. One PBS control DPP tissue sample was excluded from the analysis due to poor sample quality. Differences in cytokine transcript abundance in *in vitro* re-stimulated cells (i.e., LP, MLN, and PP cells) isolated from MAP infected (DPP, *n* = 4; CPP, *n* = 4) and PBS control (DPP, *n* = 4; CPP, *n* = 4) segments was determined using an unpaired, two-tailed Student's *t* test, unless otherwise specified, and presented as relative expression. Cells isolated from one MAP infected DPP intestinal segment were excluded from the analysis due to poor sample quality. *f57* gene copies per g of tissue were normalized using log(x) transformation and differences in copy numbers between MAP-infected DPP (*n* = 5) and CPP (*n* = 4) PP was determined using an unpaired, two-tailed Student's *t* test. CFU enumeration data was normalized using log(x) transformation. Differences in CFU counts between discrete (*n* = 5) and continuous (*n* = 5) PP tissue was determined using a paired Student's *t* test. A one-way ANOVA was applied to compare CFU counts among three independent challenge studies (*n* = 11, 17, and 18). For correlation analysis between *f57* gene copy numbers and MAP CFU counts, Pearson's correlation was used. *p* values ≤ 0.05 were considered statistically significant.

## Results

### Clinical Responses of Calves Following Surgical Isolation of Intestinal Segments

Daily monitoring of calves post-operatively revealed no changes in body temperature or clinical signs of abdominal discomfort or pain. No abnormal changes in feed intake or fecal consistency were observed throughout the 12-month study.

### Gross Appearance and Histology of Intestinal Segments

**Figure 1:**
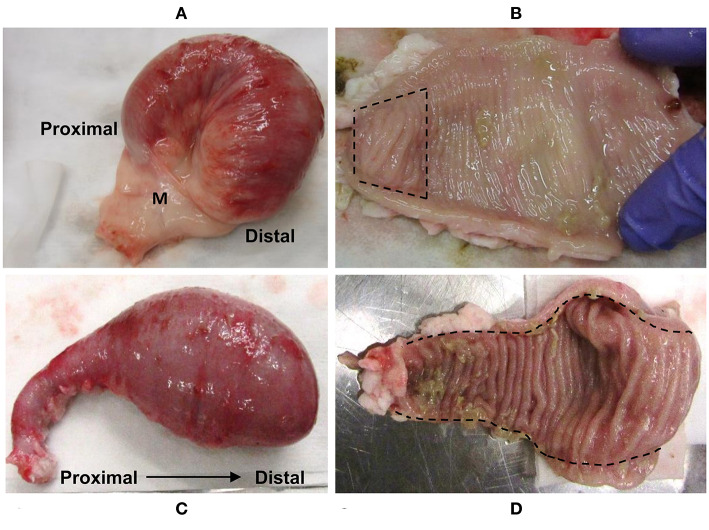
Intestinal segments removed from the abdomen at 12 months post-surgery. Representative images of the serosal surface of **(A)** mid-jejunum and **(C)** terminal jejunal segments. **(B)** Mid-jejunal segment opened along the mesenteric attachment and luminal contents removed to show mucosal surface with a DPP (between the hash marks). **(D)** Terminal jejunal segment opened along mesenteric attachment and luminal contents removed to reveal the CPP running the entire length of the segment. M, mesentery.

**Figure 2:**
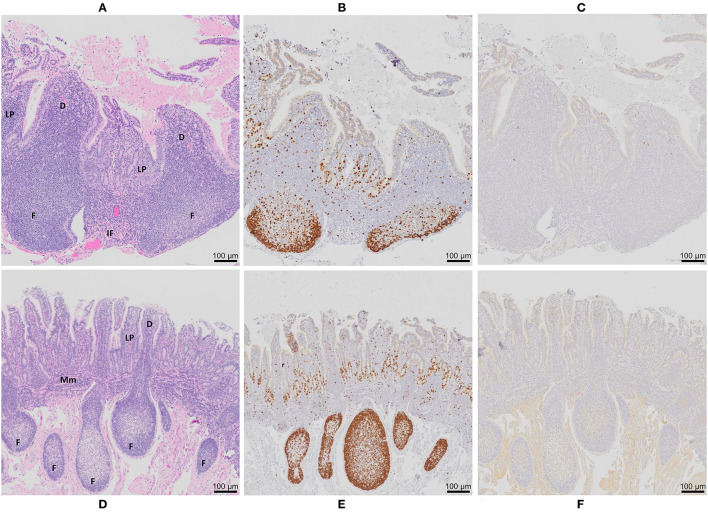
Peyer's patch architecture and histochemical staining of intestinal segments from control animals 12 months post-surgical isolation. Serial sections of **(A–C)** a DPP and **(D–F)** a CPP were stained with **(A,D)** hematoxylin and eosin, **(B,E)** Ki-67 (brown color), and **(C,F)** MAP antigen (brown color). D, dome region; F, lymphoid follicle; IF, interfollicular region; LP, lamina propria; Mm, muscularis mucosae. Magnification is 200X.

**Figure 3:**
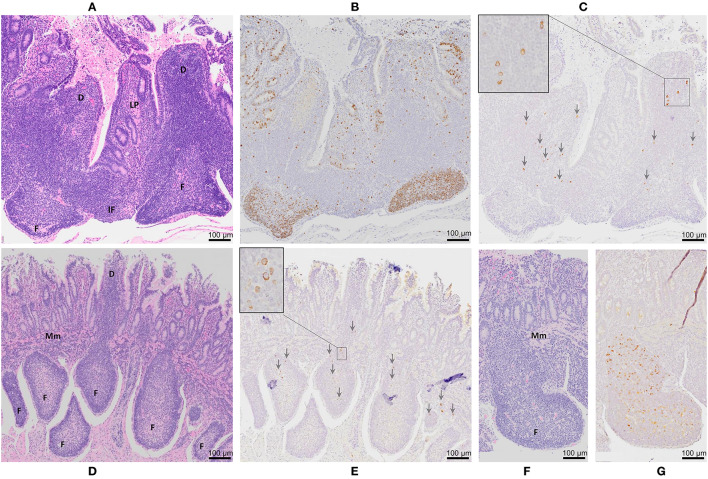
Peyer's patch architecture and histochemical staining of intestinal segments 12 months after MAP infection. Serial sections of a DPP were stained with **(A)** hematoxylin and eosin, **(B)** Ki-67 (brown color), and **(C)** MAP antigen (brown color and arrowheads). Serial sections of two CPPs stained with **(D,F)** hematoxylin and eosin and **(E,G)** MAP antigen (brown color and arrowheads). D, dome region; F, lymphoid follicle; IF, interfollicular region; Mm, muscularis mucosae. Magnification is 200X. Insets in Panels **(C,E)** are digital magnifications of boxed areas in the **(C)** dome region and **(E)** lymphoid follicle showing staining for MAP antigen occurs predominantly within the cytoplasm.

At 12 months post-surgery, the isolated intestinal segments had maintained their mesenteric attachment and the resected bowel and anastomoses appeared normal. After removal of intestinal segments from the abdomen, examination of the serosal surface revealed no visible abnormalities or discolouration when compared to adjacent intestine (Figures 1A,C). This is consistent with our previous observations of intestinal segments collected at 9 months post-surgery (35). Fibrous attachments were present on the serosal surface of some intestinal segments, resulting in localized omental adhesions. There was no evidence of luminal content leakage into the abdomen. Segments were more distended at their distal end due to the accumulation of luminal contents, consistent with ongoing peristaltic contractions in the aboral direction. After opening each segment, a dense, mucoid mass was present in the intestinal lumen and the mucosal surface was uniform in color and texture with no visible erosion or discolouration of the epithelial surface (Figures 1B,D). Further, each intestinal segment retained a visible PP. In the mid-jejunal segments, a single anti-mesenteric DPP was visible with clearly demarcated borders and was surrounded by thinner jejunal tissue (Figure 1B). An anti-mesenteric CPP with clearly demarcated borders was visible in the distal small intestine segment (Figure 1D). Histology confirmed that the anatomical structure of an organized PP had been retained (Figures 2, 3). Hematoxylin and eosin staining of formalin-fixed tissue sections revealed that both the mid-jejunal (Figure 2A) and distal small intestine (Figure 2D) segments had retained an intact epithelial barrier but villous atrophy was evident when compared to adjacent small intestine. Lymphocytes were abundant in the lamina propria (LP) beneath each villous and follicle-associated epithelium and dome regions were visible above the sub-mucosal lymphoid follicles within each PP. Small interfollicular accumulations of lymphocytes were observed in the ileal CPP, and fewer lymphoid follicles with more extensive interfollicular accumulations of lymphocytes were present in the jejunal DPPs. There was, however, a noticeable reduction in the size of lymphoid follicles when comparing PPs in the isolated intestinal segments with PP located in adjacent intestine.

Immuno-histochemical staining (IHC) of tissue sections for Ki-67 (a marker of proliferating cells) was completed to determine whether lymphoid follicles in the PPs continued to function as sites of lymphoproliferation and whether crypt epithelium retained its capacity to generate the cells required to maintain the epithelial barrier. In DPP and CPP of control (Figures 2B,E) and MAP infected intestinal segments (Figure 3B, data shown for DPP only) Ki-67 staining was most abundant within lymphoid follicles and mucosal crypts and less abundant in cells dispersed throughout dome regions and interfollicular regions. This staining pattern was consistent with that observed in PPs located in the adjacent intestine of both control and infected calves. Collectively, PPs within surgically isolated intestinal segments, with or without MAP challenge, retained an anatomical, structural and compartmental organization that was similar to the PPs located in the adjacent intestine. There were, however, visible changes in the size of lymphoid follicles and epithelial villi. There was a significant (*p* < 0.0001) reduction in the length of CPP lymphoid follicles in the isolated intestinal segments (521 ± 55 μm; mean ± 1SD) when compared to CPP follicle length (1406 ± 55 μm; mean ± 1SD) in the adjacent intestine. Lymphoid follicles were reduced to approximately one third (63% reduction) their normal length when CPP were present for 12 months within surgically isolated intestinal segments. This reduction in follicle length occurred despite evidence of persistent lymphopoiesis (Figure 2). To control for these changes, all analyses of MAP-infected segments were made relative to PBS control segments.

### Persistence of MAP in Discrete and Continuous PP

The presence of MAP within each intestinal segment was determined using qPCR to amplify the single copy MAP-specific DNA element *hspX* in formalin-fixed PP tissue and the single copy DNA element *f57* in fresh frozen PP tissue (Table 1). Control (*n* = 5) DPP and CPP tissue and MLNs draining these intestinal segments were all PCR-negative for *hspX* and *f57*. All four MAP-infected segments containing a CPP were *f57* PCR-positive, two of which were also PCR-positive for *hspX*. In contrast, only two of five MAP-infected segments containing a DPP were *f57* positive and all five were *hspX* PCR-negative. Furthermore, the mean log *f57* gene copy number/g of DPP tissue was significantly lower (*p* = 0.046) when compared to CPP tissue.

**Table 1:** Summary of study animals and infection status at 12 months post-challenge.

**Intestinal segment site and PP:**	**Challenge dose**	**Calf ID**	***f57* gene copies/g PP tissue**	***hspX* qPCR PP tissue**	***hspX* qPCR MLN**
Mid-jejunum discrete PP	1 × 10^9^ CFU	29	6.6 × 10^3^	Negative	Negative
		35	3.1 × 10^3^	Negative	Negative
		37	Negative	Negative	Negative
		42	Negative	Negative	Negative
		47	Negative	Negative	Negative
Terminal jejunum continuous PP	1 × 10^9^ CFU	28	1.2 × 10^4^	Positive	Negative
		34	1.5 × 10^4^	Negative	Negative
		36	1.7 × 10^5^	Positive	Negative
		40	7.6 × 10^2^	Negative	Negative
Mid-jejunum discrete PP	PBS	31	Negative	Negative	Negative
		33	Negative	Negative	Negative
		39	Negative	Negative	Negative
		41	Negative	Negative	Negative
		43	Negative	Negative	Negative
Terminal jejunum continuous PP	PBS	31	Negative	Negative	Negative
		33	Negative	Negative	Negative
		41	Negative	Negative	Negative
		43	Negative	Negative	Negative

*MAP bacterial burden was determined by quantifying the single copy MAP-specific DNA element f57 (55) in fresh frozen PP tissue and the single copy MAP-specific DNA element hspX (62) in formalin-fixed tissue by qPCR. Control calves had two segments surgically isolated: one in the mid-jejunum containing a DPP and the other in the terminal jejunum containing a CPP, and each injected with PBS. PP, Peyer's patch; MLN, mesenteric lymph node*.

To further validate MAP presence or absence in the DPP and CPP, tissue sections were stained with MAP antisera. PP tissue from control intestinal segments served as negative controls. A few scattered, weakly stained cells were observed in the dome regions of control DPPs (Figure 2C) but there were no visible stained cells in control CPP tissue sections (Figure 2F). No visible IHC stained cells were observed in tissue sections from *f57* PCR negative MAP-infected DPPs, but visible cellular staining was detected in one of the two *f57* PCR positive MAP-infected DPPs. Visible staining in this single DPP was localized to cells in the dome regions (Figure 3C). In contrast, IHC staining of cells was observed in all MAP-infected CPPs (Figure 3E) with cellular staining in the dome regions similar to that observed in the MAP-infected DPP. As well, abundant cellular staining in CPPs was evident within many lymphoid follicles (Figures 3E,G). IHC staining of cells was rarely observed in the interfollicular regions of PPs, lamina propria, or MLNs draining either the MAP-infected mid- or distal small intestine segments.

PCR and IHC analyses previously confirmed that MAP injected into the lumen of intestinal segments of 10–14 day old calves resulted in a consistent and persistent MAP infection in both DPP and CPP tissue at 2 months post-infection (34). We further validated the consistent uptake and persistence of MAP strain gc86 by enumerating viable bacteria recovered from both DPP and CPP at one month post-infection (Supplementary Figure 1A). No significant (*p* = 0.78) difference in viable MAP recovery was detected between DPP and CPP (*n* = 5). Moreover, no differences (*p* = 0.28) in viable MAP recovery was detected among three independent MAP infection studies (*n* = 11, 17, 18) when using surgically isolated intestinal segments (Supplementary Figure 1B). Thus, targeted delivery of MAP to intestinal segments results in reproducible and consistent MAP infection. These data suggest that MAP infection persists equally in DPP and CPP of all animals between 1–2 months post-infection, however, at l2 months post-infection there was a divergence in MAP persistence in DPP when compared to CPP.

### Transcriptome of Discrete and Continuous PP, and Draining MLN

To better understand the regional differences in MAP persistence between CPP and DPP, global transcriptomic profiling with RNA-seq was used to investigate transcriptional changes at the site of MAP infection. The transcriptome of the 12 month MAP-infected DPP (*n* = 5) and CPP (*n* = 4) were compared relative to PBS control segments (*n* = 5), as well as MLNs draining each intestinal segment. In MAP-infected DPPs 1,707 genes were differentially expressed (fold change ±1.5 fold; adjusted *p* < 0.05) when compared to uninfected DPPs (Supplementary Table 2). In contrast, only 4 genes were differentially expressed in MAP-infected CPPs (Supplementary Table 3) when compared to uninfected CPPs. No differentially expressed genes were identified in the MLNs draining MAP-infected segments containing either DPP or CPP when compared to MLNs draining PBS control segments.

**Figure 4:**
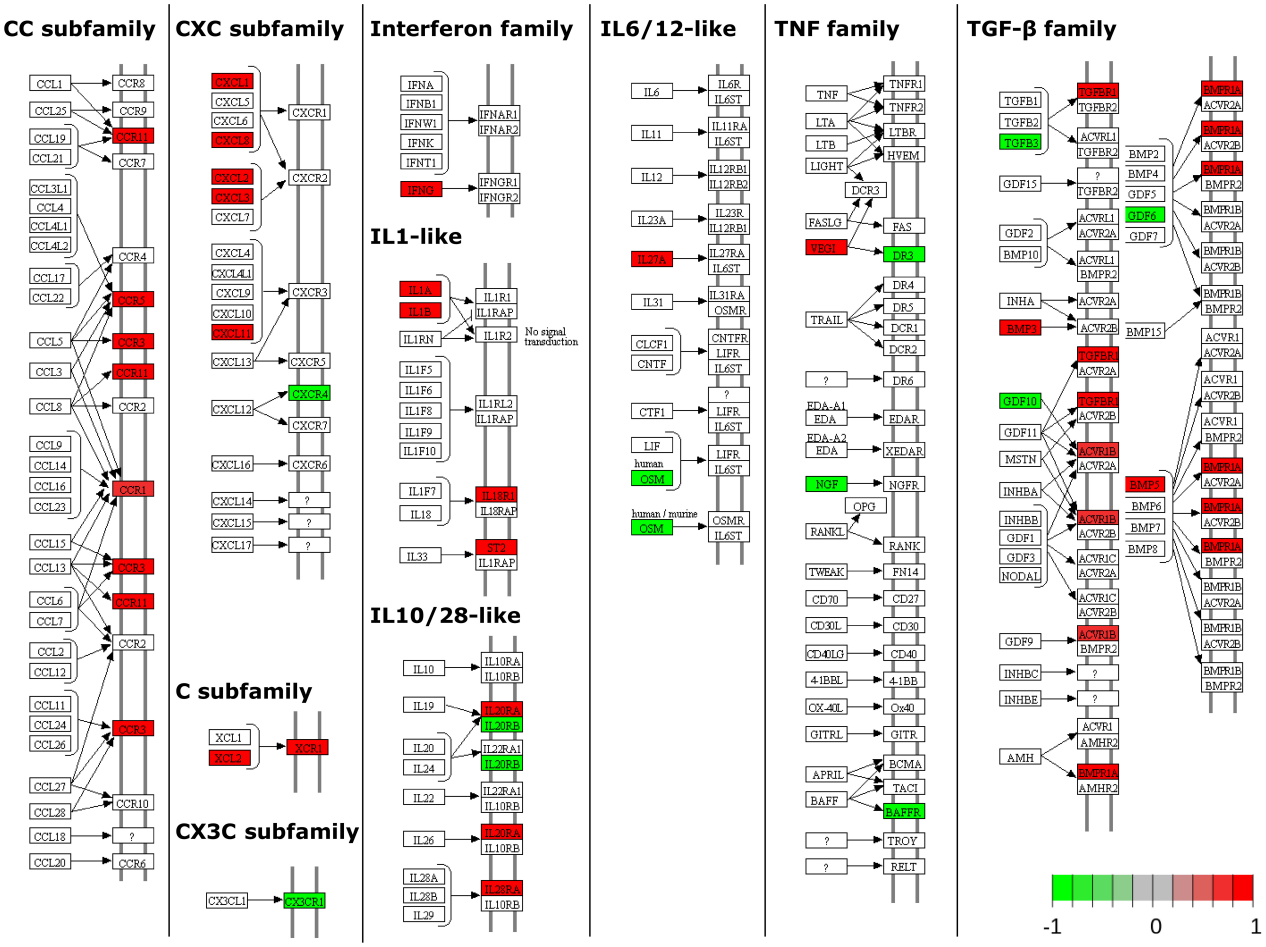
Pathway-based visualization of differentially expressed genes encoding cytokines and chemokines and their receptors when comparing MAP-infected DPP to PBS control intestinal segments. Data was rendered using Pathview (63). Red, upregulated; green, downregulated.

**Figure 5:**
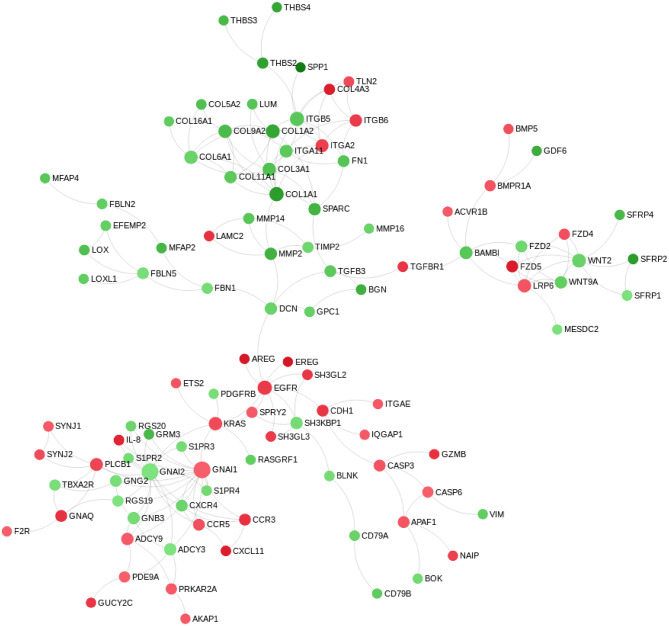
Zero-order protein-protein interaction network of differentially expressed genes comparing MAP-infected DPP to PBS control intestinal segments. Data was drawn using NetworkAnalyst (57). Red, upregulated; green, downregulated. Node size reflects the number of protein-protein interactions, with larger nodes representing a greater number of annotated interactions.

Pathway analysis, using InnateDB (56), of differentially expressed genes in MAP-infected DPPs identified two significantly upregulated pathways, including *chemokine receptors bind chemokines* and *metabolism*, and 61 significantly downregulated pathways (Supplementary Table 4). Pathview (63) (Figure 4) and NetworkAnalyst (57) zero-order protein-protein interaction network visualization (Figure 5) further highlighted a substantial number of specific chemokines (6 upregulated) and chemokine receptors (9 upregulated and 2 downregulated), cytokines (7 upregulated, 6 downregulated) and cytokine receptors (19 upregulated, 5 downregulated), immune cell surface markers (e.g., *CD79A, CD79B*) and innate immune-related genes (e.g., *CASP3, CASP6, granzyme B*) as potentially involved in the mucosal immune response to MAP. These data show that the reduction of MAP burden in DPP is associated with significant global transcriptional changes (involving a number of immune-related genes) whereas the persistence of MAP infection in CPP was associated with minimal changes to the global transcriptome. This dichotomy provides an opportunity to further investigate immune responses associated with control of MAP infection vs. persistence of infection, and to identify potential surrogate markers of immune protection.

### Cytokine Gene Expression in MAP-Infected Discrete and Continuous PPs

**Figure 6:**
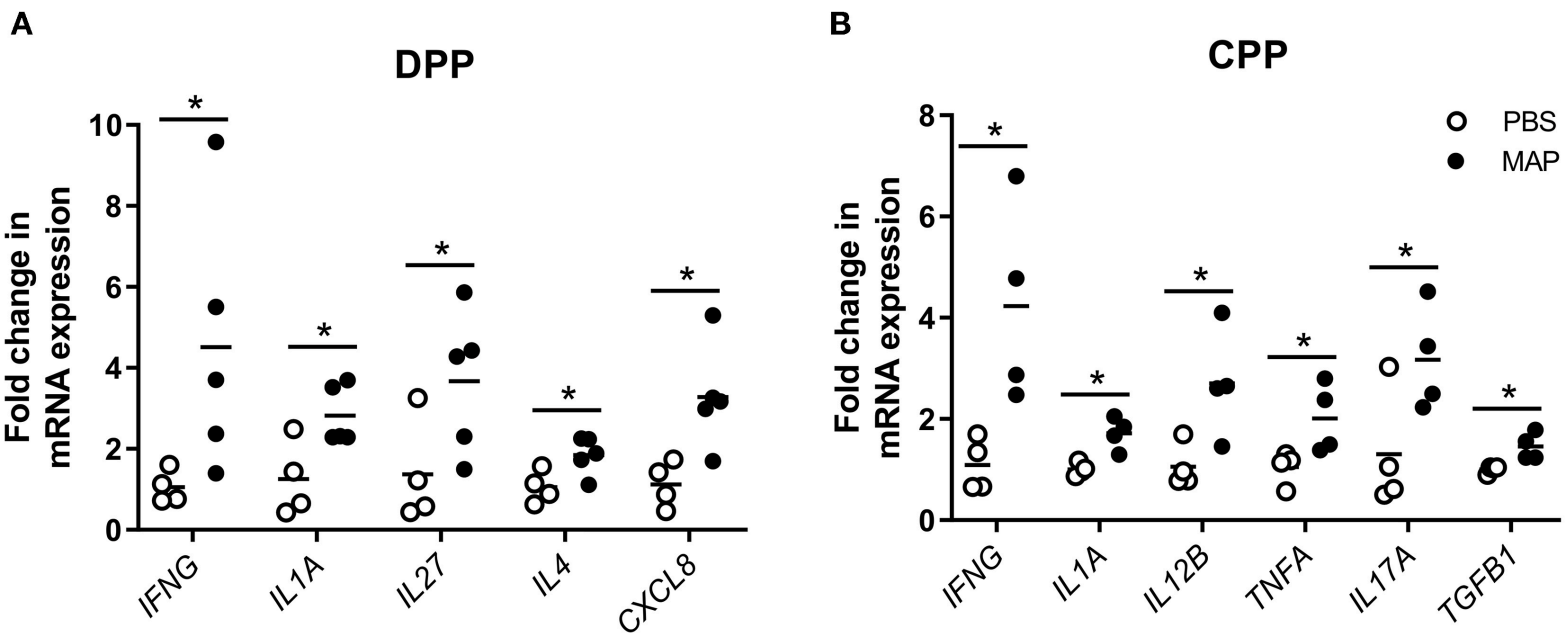
Differentially expressed cytokine genes in MAP-infected DPP and CPP at 12 months post-infection relative to PPs collected from PBS control segments. Transcript abundance for 23 cytokine genes (assessed using the primers described in Supplementary Table 1) was quantified by qRT-PCR in PP tissue from **(A)** MAP-infected (*n* = 5) and PBS control (*n* = 4) DPP segments, and **(B)** MAP-infected (*n* = 4) and PBS control (*n* = 4) CPP segments. Each data point represents the fold change for each individual animal relative to the mean of the PBS control segments. Data is presented only for cytokines showing differential (*p < 0.05) gene expression in MAP-infected segments compared to PBS control segments.

To identify specific cytokines associated with the control of MAP infection, we compared cytokine gene expression in control and MAP-infected PP tissues. Using qRT-PCR we quantified transcript abundance for 23 cytokine genes (Supplementary Table 1) associated with Th1, Th2, and Th17 responses. In MAP-infected DPPs (*n* = 5), *CXCL8, IL4*, and *IL27* were upregulated (*p* < 0.05) when compared to DPP tissue collected from PBS control segments (Figure 6A). In contrast, *IL12B, IL17A, TGFB1*, and *TNFA* were upregulated (*p* < 0.05) in MAP-infected CPPs (*n* = 4) when compared to CPP tissue collected from PBS control segments (Figure 6B). In both MAP-infected DPP and CPP, *IFNG* and *IL1A* were each upregulated (*p* < 0.05) relative to their respective PBS control segments.

**Figure 7:**
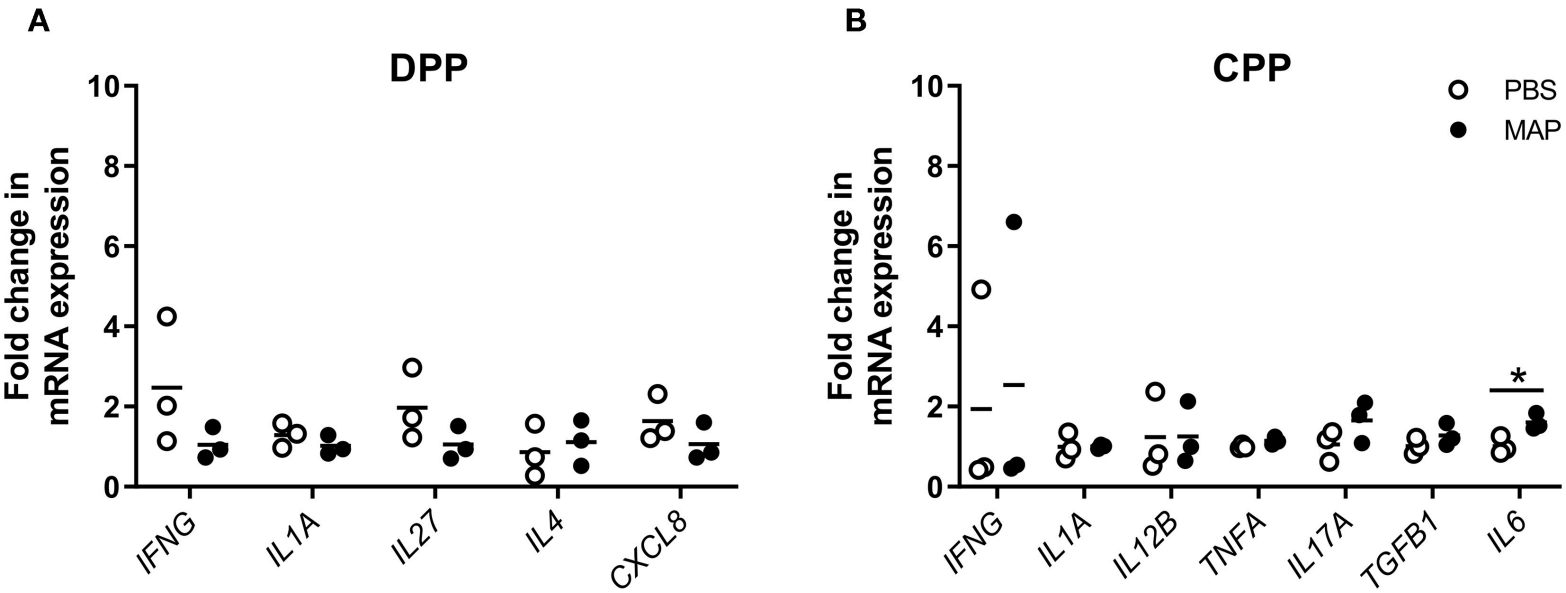
Differential cytokine gene expression in PP tissue from MAP-infected segments compared to PBS control segments at 2 months post-infection. Transcript abundance for 23 cytokine genes (assessed using the primers described in Supplementary Table 1) was quantified by qRT-PCR in **(A)** MAP-infected (*n* = 3) and syngeneic PBS control (*n* = 3) DPP tissue, and **(B)** MAP-infected (*n* = 3) and syngeneic PBS control (*n* = 3) CPP tissue collected from a previous study (34). Each data point represents the fold change for each individual animal relative to the mean of the PBS control segments. Data presented are for cytokine genes showing differential (*p < 0.05) gene expression in MAP-infected segments relative to PBS control segments at 2 months post-infection, in addition to those cytokine genes that were identified as differentially expressed at 12 months post-infection (Figure 6).

To determine whether differentially expressed cytokine genes could only be identified following a prolonged MAP infection (12 months post-infection) we also analyzed PP tissues collected at an earlier time point of 2 months post-infection. These 2 month post-infection tissue samples were obtained from surgically isolated intestinal segments prepared in neonatal calves challenged with an equivalent dose of the MAP gc86 strain as described in this study (34). Of the 23 cytokine genes analyzed, none were significantly (*p* > 0.05) differentially expressed in MAP-infected DPPs (*n* = 3) relative to syngeneic PP in PBS control segments (Figure 7A). In contrast, *IL6* was significantly but modestly upregulated in MAP-infected CPPs (*n* = 3) relative to its expression in syngeneic PP from control segments (Figure 7B). The paucity of differentially expressed cytokine genes at 2 months post-infection was consistent with our previous observation that there was no significant induction of MAP-specific IFNG-secreting cells in either DPP or CPP at 2 months post-infection (34).

**Figure 8:**
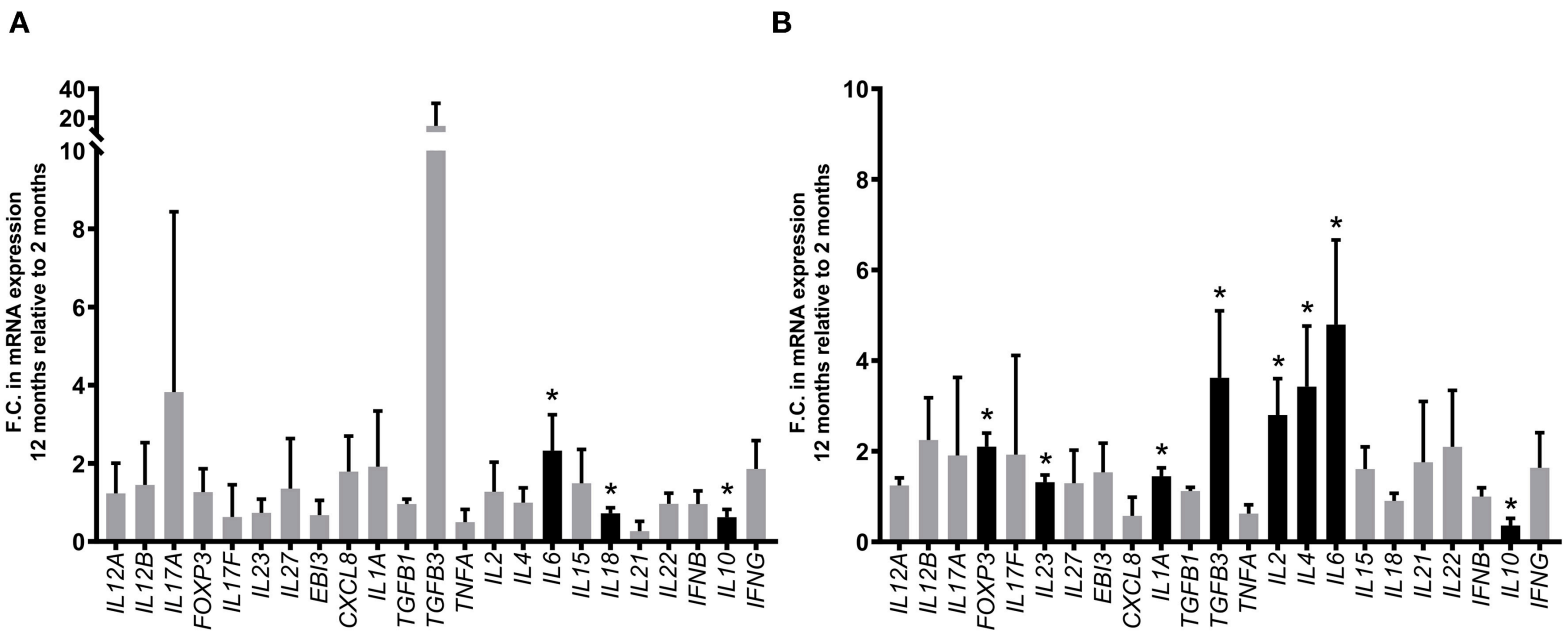
Comparison of differentially expressed cytokine genes in DPP and CPP tissue from PBS control segments at 12 months relative to 2 months post-surgical isolation. Transcript abundance for 23 cytokine genes (assessed using the primers described in Supplementary Table 1) was quantified by qRT-PCR and normalized to the constitutively expressed gene *YWHAZ*. Bars represent the mean fold change (F.C.) at 12 months post-surgery (i.e., 12-month-old calves) relative to 2 months post-surgery (i.e., 2-month-old calves) in **(A)** DPP tissue (*n* = 4 and *n* = 3, respectively) and **(B)** CPP tissue (*n* = 4 and *n* = 3, respectively). *p < 0.05, black bars. Error bars represent standard deviation.

We further investigated whether differentially expressed cytokine genes identified at 12 months post-infection might have been influenced by developmental changes in PP gene expression (64) since the CPP begins to involute at the time of sexual maturity (65). Expression of the 23 cytokine genes analyzed was compared in PPs collected from PBS control segments at 2 and 12 months post-surgery. Three of the 23 cytokine genes analyzed (*IL6, IL10, IL18)* displayed significant (*p* < 0.05) age-dependent differential expression in DPPs (Figure 8A) and 8 cytokine genes (*FOXP3, IL1A, IL2, IL4, IL6, IL10, IL23, TGFB3*) displayed significant (*p* < 0.05) age-dependent differential expression in CPP (Figure 8B). Thus, with the possible exception of *IL1A*, which displayed an age-dependent upregulation in CPP, none of the MAP-specific differentially expressed cytokine genes identified by qRT-PCR at 12 months post-infection displayed significant age-dependent changes in transcript abundance.

### Cytokine Responses Elicited by MAP Antigen Re-stimulation of Intestinal Immune Cells

**Figure 9:**
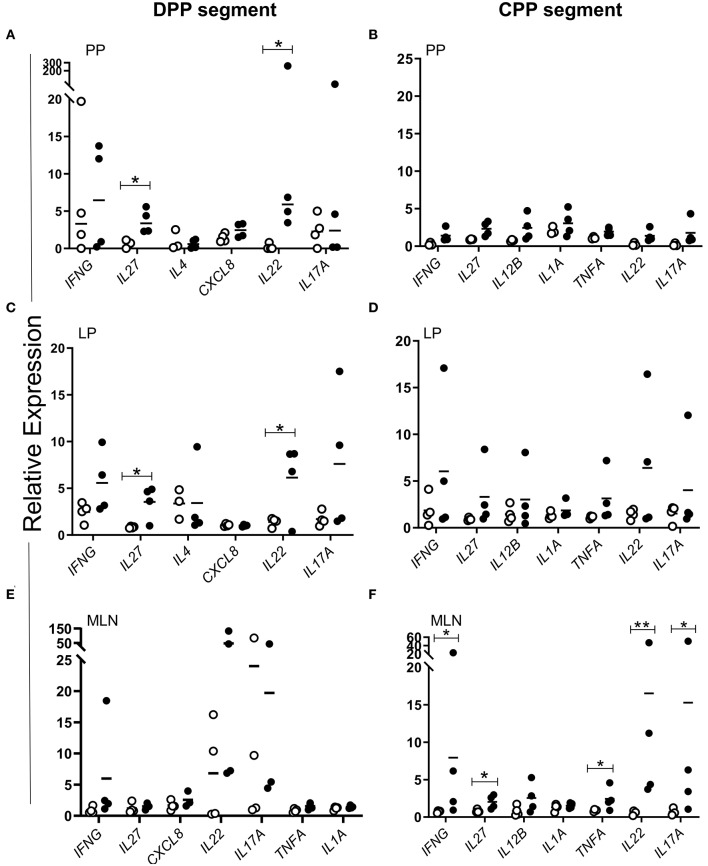
MAP-specific cytokine responses show marked regional and compartmental differences in mucosal immune response to persistent MAP infection. Freshly isolated **(A,B)** PP and **(C,D)** LP cells from MAP-infected (black dots) DPP (*n* = 4) and CPP (*n* = 4) segments and PBS control (white circles) DPP (*n* = 4) and CPP (*n* = 4) segments, and **(E,F)** cells from the draining MLN were stimulated *in vitro* with MAP whole-cell lysate (1 μg/mL; 2 × 10^6^ cells). Cytokine transcript was quantified in resting and stimulated cells to determine relative expression. Each data point represents the relative expression for each individual animal. *p < 0.05, **p < 0.01. *p* values were calculated using Student's *t* test for all datasets with the exception of *IL22* in PP cells in which a Mann-Whitney test was applied. PP, Peyer's patch; CPP, continuous PP; DPP, discrete PP; LP, lamina propria; MLN, mesenteric lymph node.

We next determined whether MAP antigen could elicit differential expression of any of the cytokine genes identified in PP tissue at 12 months post-infection in cells isolated from the LP, PP tissue and MLN draining individual intestinal segments and subsequently re-stimulated *in vitro* with MAP whole cell lysate. Cytokine gene expression in cells isolated from MAP-infected intestinal segments was compared to cells isolated from PBS control segments. We observed that whole cell lysate induced significant (*p* < 0.05) antigen-specific *IL22* and *IL27* recall responses in PP cells (Figure 9A; by 6- and 4- fold respectively) and LP cells (Figure 9C; by 6- and 3- fold, respectively) isolated from MAP-infected DPP segments. In contrast, there were no significant (*p* > 0.05) antigen-specific cytokine responses observed with PP or LP cells isolated from MAP-infected CPP segments (Figures 9B,D). In contrast, whole cell lysate stimulation of cells isolated from MLN draining the MAP-infected CPPs displayed significant (*p* < 0.05) changes in the expression of *IL22, IL27, IFNG, IL17A*, and *TNFA* (Figure 9F), but no significant (*p* > 0.05) induction of cytokine genes was detected in cells isolated from MLN draining MAP-infected DPPs (Figure 9E). Thus, *in vitro* analyses of MAP-specific responses confirmed that persistent MAP infection of both DPP and CPP resulted in regionally specific PP cytokine responses and differential cytokine responses in draining MLNs of intestinal segments containing either an infected DPP or CPP.

## Discussion

We previously demonstrated equivalent uptake and persistence of MAP infection in DPP and CPP at 2 months post-infection by IHC staining for MAP antigen and detection of the single copy DNA element *hspX* (34). To further validate this observation, recovery of viable MAP performed in the current study confirmed that all neonatal calves were infected with similar levels of MAP in DPP and CPP at one month post-infection (Supplementary Figure 1A). PCR coupled with IHC has proven more sensitive, at times, than *in situ* acid-fast staining or bacterial culture in detecting MAP in tissues, specifically at later time points post-infection (30, 31, 36, 48). Therefore, we used IHC staining of tissue in combination with PCR for detection of MAP in this study (Figures 2, 3). At 12 months post-infection, MAP DNA was detected by qPCR in only two of the five DPPs but consistently detected in CPPs (Table 1). Moreover, *f57* gene copy numbers were significantly less in MAP-infected DPP than CPP tissue by qPCR. IHC staining validated MAP infection in both DPP and CPP that were PCR positive, and further revealed the burden of infection was greater in CPP than DPP as abundant intracellular MAP staining was observed in CPP lymphoid follicles (Figure 3G) but not in DPP lymphoid follicles (Figure 3C). Thus, our challenge model consistently shows persistent MAP infection in CPP at one (Supplementary Table 1A), two (34), and 12 months post-infection. Conversely, qPCR and IHC results support the conclusion that MAP infection in DPP is more effectively controlled at 12 months post-infection relative to CPP following comparable levels of initial tissue uptake (Supplementary Figure 1A). This conclusion is further supported by histopathological analysis of intestinal tissue from naturally infected cows where fewer lesions, and less inflammation and mucosal thickening are observed in jejunal tissue (the site of DPP) when compared to ileal tissue (the site of CPP) (48).

MAP infection of DPP is not unique to our challenge model but has been confirmed by histopathology, bacterial culture and/or detection of MAP DNA in both naturally-infected calves, bulls and cows (47, 48), and following oral inoculation (30, 31, 36, 49–51). Host-pathogen interactions in DPPs have received little attention in cattle but DPPs have been recognized as an important site of infection in goats and sheep (38–40). What is intriguing about our data is that it demonstrates for the first time that reduction in MAP infection in DPPs at 12 months post-infection is associated with a widespread transcriptional response involving cytokine, chemokine and metabolism genes. In contrast, persistence of MAP infection in the CPP was associated with minimal perturbation of the global transcriptome as assessed by RNA-seq analysis. Collectively, the sustained paucity of a local host response to MAP infection in CPP at 12 and 2 months (34) post-infection argues that an unperturbed CPP might contribute to the prolonged asymptomatic stage of infection (11) and moreover, provides a source of MAP for fecal shedding facilitating horizontal transmission among calves (14, 15). Involution of the CPP at sexual maturity (65) eliminates this unique portal of entry and the lymphoid follicles within the terminal small intestine which harbor MAP (34) (Figure 3G and Supplementary Figure 1) and provide a refuge from host adaptive immune defenses. To maintain infection in older animals, MAP must be able to survive in the adjacent intestinal tissue where effector immune cells are much more abundant (61, 66), and there is a greater capacity for the induction of mucosal immune responses (34).

Our previous analysis of MAP-specific antibody responses at 2 months post-infection identified a regional dichotomy with the induction of IgA responses in DPPs but not CPPs. Although we detected MAP-specific IgA B cells in DPPs at 2 months post-infection (34), targeted qRT-PCR analysis of 23 cytokine genes associated with Th1, Th2, and Th17 responses (Supplementary Table 1) revealed no differentially expressed cytokine genes at 2 months post-infection (Figure 7A). We previously observed no detectable induction of a MAP-specific immune responses in CPP at 2 months post-infection when quantifying IgA-, IgG-, and IFNG-secreting cells (34) and in this study we provide additional evidence that only one (*IL6*) of 23 cytokine genes was modestly upregulated in CPP at 2 months post-infection (Figure 7B). The absence of a detectable immune response in CPPs is consistent with previous infection studies investigating host responses up to 12 h post-infection in ligated ileal loops (32) as well as studies performed 1-month post-infection in surgically isolated segments containing a CPP (64). Both groups identified few differentially expressed genes (<10) through transcriptomic profiling. Thus, MAP infection of both DPP and CPP, induces few transcriptional changes in immune-related genes at 2 months post-infection and the induction of MAP-specific antibody responses in DPP did not correlate with reduced infection. Transcriptomic profiling of DPPs at 2 months post-infection is warranted and may reveal significant changes in host responses beyond the 23 cytokines we assayed.

The dichotomy in host responses to MAP infection in DPP vs. CPP was prominent at 12 months post-infection, and more importantly this dichotomy correlated with reduced MAP burden in DPPs. RNA-seq analysis revealed 1,707 differentially expressed genes in MAP-infected DPPs but only 4 genes with altered expression in CPPs (Supplementary Tables 2, 3). The paucity of differentially expressed genes, specifically immune-related genes, in MAP-infected CPPs is consistent with transcriptomic profiling of ileal tissues collected from naturally infected, subclinical cows such as that of the ileocecal valve identifying 230 dysregulated genes (67), however few of these genes and none of the altered pathways were immune related. Further investigation into cytokine gene expression by qRT-PCR in this study showed 6 cytokine genes (*IFNG, IL1A, IL12B, IL17A, TGFB1, TNFA*) were upregulated in MAP-infected CPPs (Figure 6B). Previous analyses of MAP whole cell lysate stimulated immune responses at 6–15 months post-infection have consistently identified one or more of IFNG, IL12, TNFA, and/or IL17A (either transcript or protein) as up-regulated in either CPPs (68, 69) or the draining MLN cells (33, 35). Whole cell lysate-mediated stimulation of cells isolated from ileal MLNs of subclinical, naturally MAP-infected cows also induced increased secretion of IFNG, IL17A, and TNFA (70). Thus, data from our intestinal segment challenge model are consistent with data from other experimental challenge models (e.g., oral-challenge, ligated loops, cannulation) and naturally infected animals. Furthermore, these findings provide a consensus regarding altered cytokine expression, specifically those classically regarded as pro-inflammatory, as associated with MAP persistence in CPP but not associated with protective immunity. Despite the upregulation of these cytokines suggesting an active pro-inflammatory response the RNA-seq findings would argue that these genes have limited impact on the global host response in CPP. Moreover, the lack of MAP antigen-specific recall responses in isolated mucosal immune cells further substantiates the inability of the CPP to mount a robust local adaptive immune response to MAP during the early stages of infection. Collectively, the paucity of local host responses in CPP is consistent with the function of the CPP as a primary lymphoid tissue (45) and the lack of CD4 T cells in CPP lymphoid follicles (41). This lends further support to the conclusion that MAP may exploit the lymphoid follicles of CPP (Figure 3) as an immune privileged site to avoid induction of host adaptive responses.

MAP whole cell lysate induced responses in LP, MLN, and PP cells revealed further regional and compartment differences in immune responses to MAP infection (Figure 9) with novel cytokine genes associated with MAP control. Specifically, *IL22* (which has an important role in host defenses at mucosal surfaces) and *IL27* (involved in Th1 induction and in inhibiting Th17 development) were significantly (*p* < 0.05) induced by MAP whole cell lysate-mediated stimulation of cells collected from MAP-infected DPP (Figures 9A,C), but not MLNs draining these segments (Figure 9E). In contrast, *IL22* and *IL27* were not induced following MAP whole cell lysate stimulation of cells isolated from MAP-infected CPP (Figures 9B,D). These data, together with cytokine gene profiling (Figure 6) and transcriptomic profiling (Supplementary Tables 2, 3) of whole tissues support the conclusion that following a prolonged host-pathogen interaction a partially protective immune response to MAP developed in DPPs but not the CPP.

The upregulation of *IL22* and *IL27* has not been previously reported in the context of an enteric MAP infection. IL27 has previously been implicated in immunity against viral, bacterial, and parasitic diseases (71, 72) and in many reported studies was essential for limiting immune pathology (73). The pleotropic nature of IL27 has led to conflicting reports regarding its role in the context of infectious diseases (74, 75). Most investigations of IL27 have been performed in humans or mice, and reports on the role of IL27 in intestinal immunity, specifically in ruminants, is limited. In mice, deletion of *IL27* improved clearance of *Mycobacterium tuberculosis* (MTB) from the lungs (76) but subsequently lead to earlier mortality when compared to WT mice due to IL-17A-induced immunopathology (77, 78). These observations suggested a role for IL27 in regulating responses by modulating Th17 cell activity (79). Following *Mycobacterium bovis* (bTB) infection of cattle, increased lung immunopathology correlated with increased *IL17A* and *IFNG* recall responses in PBMCs (80) suggesting that IL27 might play a similar, yet unstudied, role in balancing immune protection and controlling IL17A induced pathology in cattle. *In vivo* studies focused on CD8 T cells revealed an emerging role for IL27 as an essential third activation signal. Mouse studies demonstrated that IL27 promotes CD8 T cell expansion and IFNG production during MTB infection (81) as well as influenza virus, Sendai virus, and *Toxoplasma gondii* infections (72), and has a critical role in sustaining antigen-specific CD8 T cell survival during chronic viral infections (82). The latter study identified IL27 signaling via STAT1 and transcription factor IRF1 as essential for prolonging CD8 T cell survival. Our transcriptomic profiling of MAP-infected DPP tissue revealed increased transcription of *IL27, STAT1*, and *IRF1*, warranting further investigation of this pathway as a putative mechanism of MAP control. Lastly, transcriptomic profiling of *in vitro* stimulated PBMCs revealed *IL27*, in addition to other Th17-related cytokines, as the most highly upregulated gene following bTB infection (80). Collectively, these findings suggest that diverse hosts invoke IL27 in their immune response to pathogenic *Mycobacterium* species. In our study of local immune responses, the induction of *IL27* did not correlate with decreased *IFNG* or *IL17A* (Figures 6A, 9). On the contrary, MLN cells re-stimulated *in vitro* with MAP whole cell lysates increased *IL27* expression concomitantly with *IFNG* and *IL17A* (Figure 9F). However, it remains to be determined whether this increased gene expression had an impact at the level of translation, and subsequently an immune-modulatory or -stimulatory role. Overall, further investigation of IL27 is warranted to determine the importance of its role in enteric MAP infection and protective mucosal immunity.

IL22 studies in cattle are limited but this cytokine has received much attention in TB research for its role in mediating protective immunity (83). In cattle, *IL22* is expressed in both αβTcR and γδTcR T cells (84). IL22 has been implicated as correlate of immune protection for bTB, and MTB infection in humans and mice. In mice, NK cells and IL22 were essential for BCG vaccine-induced immunity that reduced pathogen load in the lung, promoted local effector and memory CD4 T cell responses and the induction of IFNG (85). In humans, anti-MTB therapy that succeeded in reducing MTB in sputum correlated with antigen-specific induction of IL22 in PBMCs (86). In bovine TB challenge studies, transcriptomic profiling of stimulated PBMCs from BCG vaccine-protected vs. BCG vaccine-unprotected calves revealed *IL22* as the most highly upregulated gene and demonstrated that its presence correlated with increased protection (decreased pathology and bacterial burden) (87). In contrast, *IFNG* expression did not correlate with protection (87). Similar to our findings, *IFNG* did not correlate with control of MAP infection as it was modulated in both DPP and CPP. Furthermore, re-stimulation with MAP whole cell lysates did not significantly induce *IFNG* gene expression in cells isolated from MAP-infected DPPs (Figure 9). In contrast, MAP whole cell lysate induced antigen-specific recall of *IL22* with cells isolated from MAP-infected DPPs and MLNs draining CPPs. Upregulation of *IL22* occurred in tissues where MAP could no longer be detected (Figure 9). Interestingly, *IL27* and *IL22* were co-expressed within the same cell populations (Figure 9) after re-stimulation with whole cell lysate. Further work is needed to address whether both are co-translated within the same cells. These initial associations suggest that, in bovine PP, *IL22* and *IL27* may be surrogate markers of immune protection. Further work is needed to confirm whether *IL22* and *IL27* in fact play a critical role in local protective immunity and/or whether increased expression of these cytokines in CPP would similarly be associated with a protective host response.

We report for the first time a quantitative analysis of mucosal immune responses to persistent MAP infection in the two structurally and functionally distinct PP present in the small intestine of young calves. MAP infection of bovine DPPs has largely been ignored but our findings address this knowledge gap and reveal: (i) MAP similarly infects jejunal DPP and ileal CPP; (ii) DPPs can function as an immune induction site where protective mucosal immune responses can limit MAP infection over a 12 month period, and (iii) CPPs failure to control MAP infection is associated with a paucity of pathogen-specific immune responses. Transcriptomic analyses of the marked differences in host responses occurring at these two intestinal sites enabled us to identify novel cytokines, including both *IL22* and *IL27*, associated with the control of MAP infection in DPPs. However, further work is needed to identify which effector cell populations in DPP are responsible for IL22 and IL27 production, as well as to define the role these effector cells and cytokines play in immune protection during MAP infection. These analyses will further our understanding of both mechanisms mediating control and persistence of MAP infection in the natural host and potentially guide the design and selection of effective therapeutics, such as vaccines.

## Data Availability Statement

All datasets generated for this study are included in the article/Supplementary Material. The RNA-seq datasets for this study have been deposited at the public repository NCBI Gene Expression Omnibus under Accession Number GSE141962.

## Ethics Statement

This study was carried out in accordance with the principles of the Basel Declaration and recommendations of The Guide to the Care and Use of Experimental Animals, Canadian Council on Animal Care. The protocol was approved by the University of Saskatchewan Animal Care Committee.

## Author Contributions

AF, LM, and PJG designed the study. AF, AL, HT, RH, and PJG contributed to data analysis and data interpretation. AF, AL, PG, and RF performed the experiments. AF, AL, RH, and PJG wrote the manuscript. HT, VG, AP, and SN contributed to manuscript preparation.

## Conflict of Interest

The authors declare that the research was conducted in the absence of any commercial or financial relationships that could be construed as a potential conflict of interest.
