# Supplementary figures and images for: Regional Dichotomy in Enteric Mucosal Immune Responses to a Persistent Mycobacterium avium ssp. paratuberculosis Infection
Source: Front Immunol. 2020 May 29;11:1020. doi: 10.3389/fimmu.2020.01020 (PMC7272674; doi:10.3389/fimmu.2020.01020)

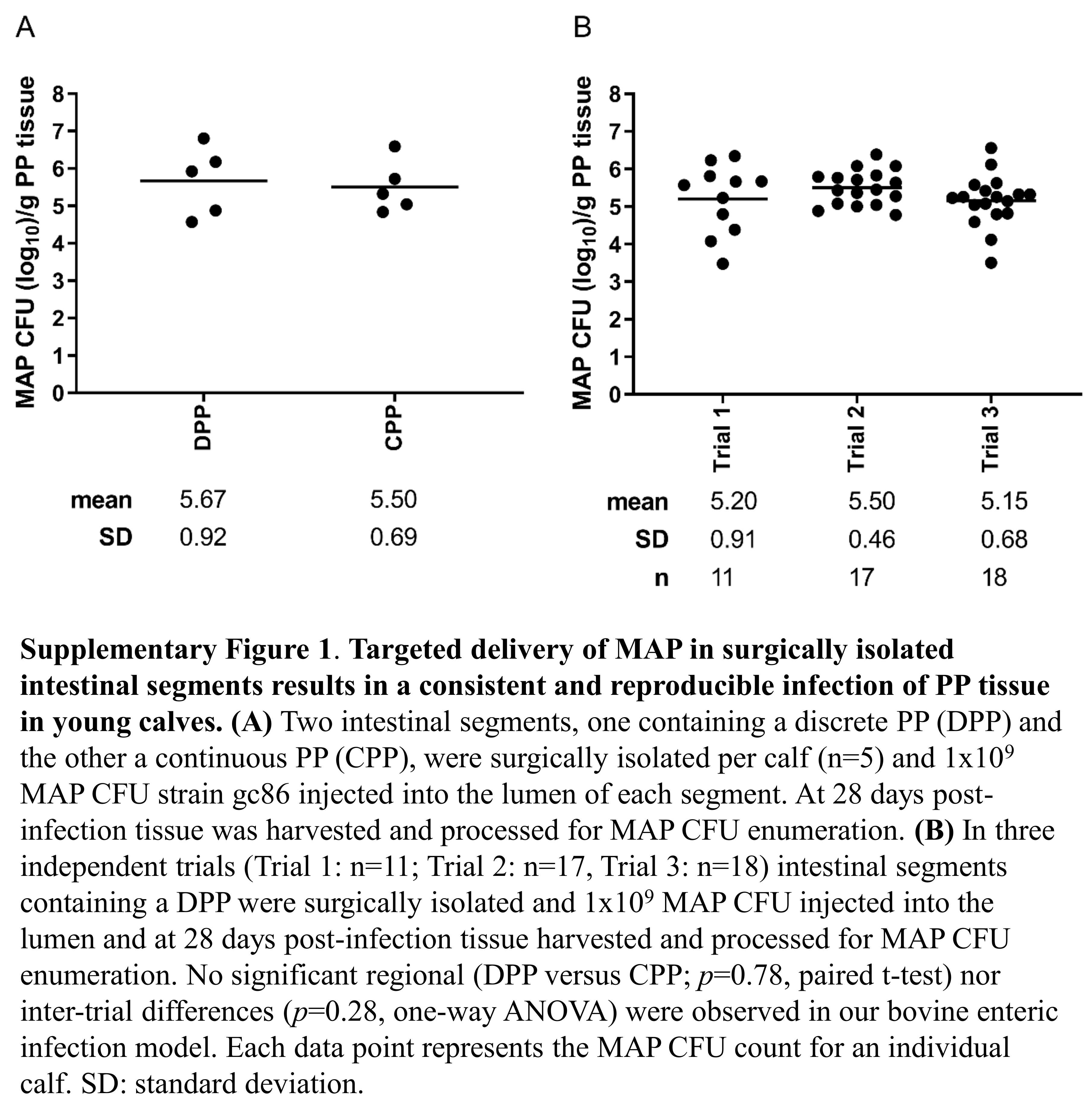

Supplement: Supplementary file 5 [file Image_1.TIF]

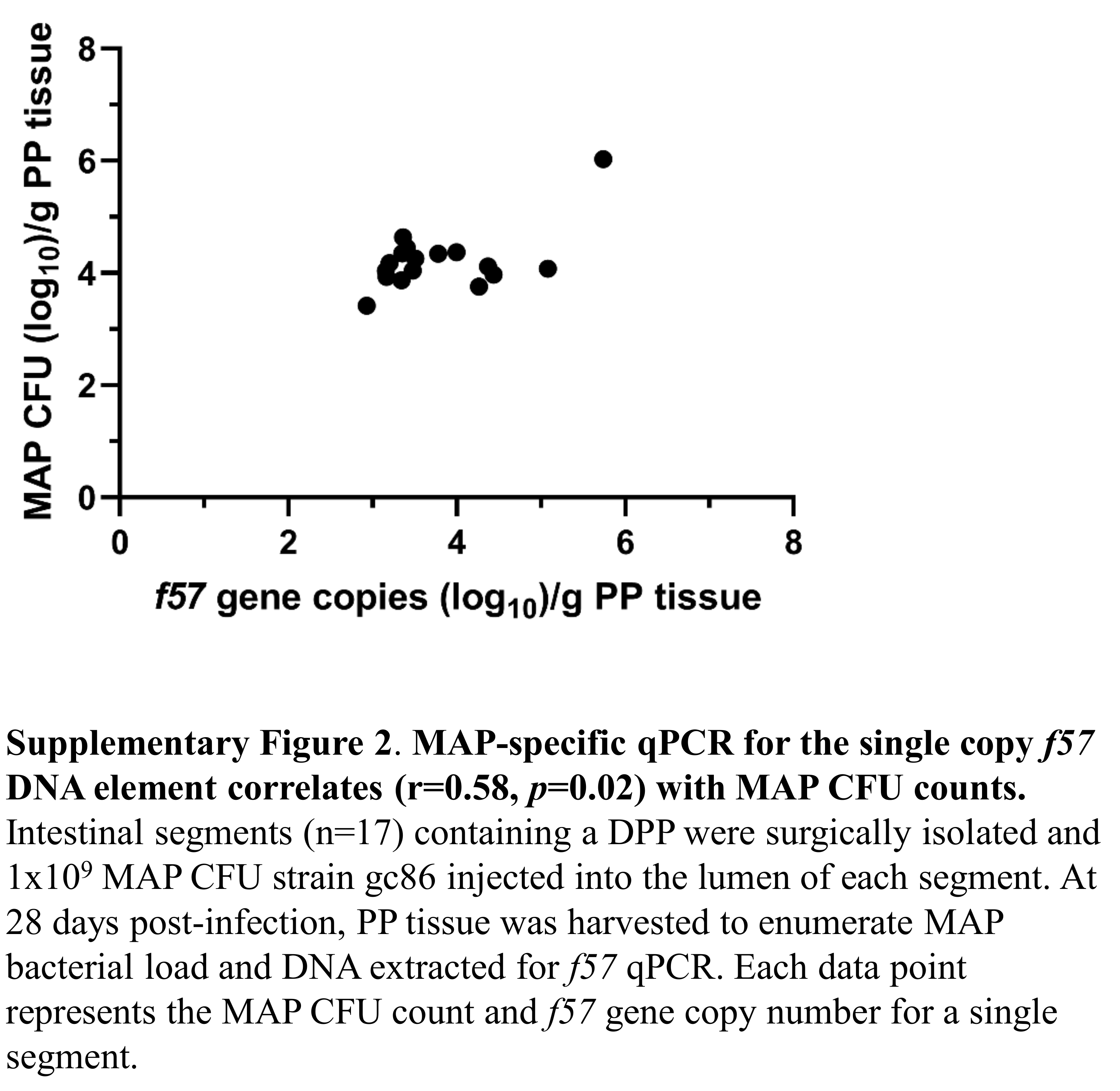

Supplement: Supplementary file 6 [file Image_2.TIF]
